# Gestational weight gain targets during the second and third trimesters of pregnancy for women with gestational diabetes mellitus in China

**DOI:** 10.1038/s41430-018-0358-9

**Published:** 2018-10-30

**Authors:** Jiang-Nan Wu, Wei-Rong Gu, Xi-Rong Xiao, Yi Zhang, Xiao-Tian Li, Chuan-Min Yin

**Affiliations:** 1 Department of Clinical Epidemiology, Obstetrics and Gynecology Hospital of Fudan University, Shanghai, 200011 China; 2 Department of Obstetrics, Obstetrics and Gynecology Hospital of Fudan University, Shanghai, 200011 China; 3 Department of Clinical Epidemiology, Children’s Hospital of Fudan University, Shanghai, 200025 China; 4 Department of Nutrition, Obstetrics and Gynecology Hospital of Fudan University, Shanghai, 200011 China

**Keywords:** Weight management, Preventive medicine

## Abstract

**Background/objectives:**

Gestational weight gain (GWG) recommendations for pregnant women with gestational diabetes mellitus (GDM) in China are lacking. The present study aims to examine whether specific GWG targets for women with GDM can improve pregnancy outcomes in comparison with GWG according to the Institute of Medicine (IOM) targets.

**Subjects/methods:**

Pregnant women diagnosed with GDM were selected from a retrospective cohort study of 8299 singleton pregnant women aged 18–45 years in 2012 (*n* = 1820). GWG ranges were calculated using a receiver operating characteristic (ROC) curve analysis (ROC targets) and the interquartile range (IR) method (the range from the 25th to 75th percentiles of the GWG among GDM women without adverse pregnancy outcomes, IR targets).

**Results:**

The incidences of small for gestational age (SGA) births and pregnancy hypertension among women with GDM who gained weight within the ROC targets were lower than the incidences in women who gained weight within the IOM targets (SGA, 7.5% vs. 8.6%; pregnancy hypertension, 12.6% vs. 14.1%; both *P* < 0.05). GWG was associated with a risk of adverse pregnancy outcomes in the total sample (estimated values ranged from −2.95 to 2.08, all *P* < 0.05). No statistically significant associations between GWG and adverse pregnancy outcomes were observed in subgroups of pregnant women with appropriate GWGs according to the ROC, IR, and IOM targets. The ROC targets exhibited higher negative predictive values for adverse pregnancy outcomes than the IR and IOM targets.

**Conclusion:**

The ROC targets improved pregnancy outcomes and thus represent potential special GWG guidelines for women with GDM in China.

## Introduction

Gestational diabetes mellitus (GDM) is defined as “diabetes diagnosed in the second or third trimester of pregnancy that is not clearly overt diabetes,” and it is a risk factor for maternal and perinatal complications [1, 2]. The global prevalence of GDM is increasing in parallel with the increase in the prevalence of overweight and obesity among pregnant women, and it is correlated with physical inactivity, dietary patterns, and the sociodemographic characteristics of pregnant women (e.g., advanced maternal age and ethnicity) [3–6].

In China, the prevalence of GDM has rapidly increased since new diagnostic criteria were recommended [7–9]. Based on the new criteria, the prevalence of GDM is ∼10% of pregnancies in municipalities, such as Beijing and Tianjin [10, 11]. In Shanghai, more than 20% of pregnant women were diagnosed with GDM in 2012 based on our previous study of 8299 singleton pregnant women [12].

Inappropriate gestational weight gains (GWGs), including excessive or insufficient GWGs, are associated with an increased risk of adverse pregnancy outcomes [13–16]. The Institute of Medicine (IOM) published recommendations for GWG in 1990 and revised them in 2009 [17, 18]. The IOM GWG targets are intended for women of all ethnicities and statures and are frequently applied to a specific population, such as women with GDM [18, 19]. However, researchers have not determined whether the IOM targets are applicable to women with GDM who have a greater underlying risk of adverse outcomes.

In a retrospective cohort study of pregnant Australian women with GDM, Wong et al. found that GWG defined according to modified IOM targets did not improve prenatal outcomes among pregnant women with GDM [19]. However, the authors simply subtracted an unwarranted 2 kg from the upper IOM target or from both the upper and lower targets or copied the range (0–4 kg) for women with a body mass index (BMI) ≥ 35 kg/m^2^ from another study [19, 20]. These methods for modifying the GWG targets might reduce the reliability of the effect of the targets on adverse pregnancy outcomes. Moreover, the body composition and dietary habits of pregnant Chinese women differ from those of pregnant women from Western countries, indicating that GWG targets differ between Eastern and Western women with GDM [21, 22]. Nevertheless, no studies have been conducted to determine the GWG ranges for Chinese women with GDM.

We conducted a retrospective cohort study of singleton pregnant women with GDM to determine GDM-specific GWG targets for pregnant Chinese women. Because Chinese women are more likely to develop GDM than other populations [23] and women with GDM have a relatively high risk of adverse pregnancy outcomes [19, 24], we hypothesize that GDM-specific GWG targets may improve pregnancy outcomes.

## Methods

### Subjects and data collection

This report is part of a retrospective cohort study of 8299 singleton pregnant women aged 18–45 years who received prenatal examinations and care at the Obstetrics and Gynecology Hospital of Fudan University between January 1 and December 31, 2012. The retrospective cohort study was conducted between 2013 and 2015 to estimate the prevalence of GDM among pregnant women in Shanghai, and was restricted to a review of pregnant women in 2012 because of the time and funding constraints. An oral glucose tolerance test (OGTT) was administered to all pregnant women at 24–28 weeks of gestation to detect GDM. Pregnant women with a singleton pregnancy who were diagnosed with GDM were included in this study. Exclusion criteria were women with multiple pregnancies and a lack of data on the GDM diagnosis or pregnancy outcomes.

Baseline information, including the maternal age, pre-pregnancy body weight, height, and parity, was surveyed, and the blood pressure and levels of the fasting blood glucose, triglyceride, cholesterol, high-density lipoprotein, low-density lipoprotein, and DII dimer were measured at the first visit to the hospital. Plasma glucose levels were measured using enzyme-linked colorimetry, triglyceride levels were measured using a free glycerol method, and cholesterol levels were measured using a cholesterol oxidase method. The direct method was used to measure levels of high- and low-density lipoproteins. Levels of the DII dimer were measured using a turbidimetric inhibition immunoassay. Body weight and blood pressure were measured for all subjects at each visit. The mean number of visits was 7.9 (standard deviation, SD, 1.9) and ranged from 2 to 15 visits. Body weight, gestational weeks at labor and birth outcomes (birth weight and Apgar scores at 1 and 5 min after birth) were recorded after delivery. These data were extracted from the electronic medical record systems maintained by the hospital.

### Definitions of outcomes and variables

Weekly GWGs measured during the second and third trimesters were calculated as the GWG from the 12th week of pregnancy until delivery divided by the number of gestational weeks in the second and third trimesters (weeks at labor minus 12 weeks). Pre-pregnancy BMI was calculated as [pre-pregnancy body weight (kg)]/[height^2^ (m^2^)]. According to the pre-pregnancy BMI classification standard for Chinese adults [25], pregnant women were classified into the following categories: underweight (BMI < 18.5 kg/m^2^), normal weight (BMI 18.5–23.9 kg/m^2^), overweight (BMI 24.0–27.9 kg/m^2^), or obese (BMI ≥ 28 kg/m^2^). We also used pre-pregnancy BMI classifications based on the IOM standard to classify the women into the following groups: underweight (BMI < 18.5 kg/m^2^), normal weight (BMI 18.5–24.9 kg/m^2^), overweight (BMI 25.0–29.9 kg/m^2^), and obese (BMI ≥ 30 kg/m^2^) [18].

GDM was defined based on a fasting blood glucose level (BGL) ≥ 5.1 mmol/L, a 1-h BGL ≥10.0 mmol/L, or a 2-h BGL ≥ 8.5 mmol/L after a 75 g OGTT [26]. The adverse pregnancy outcomes in this study included premature delivery, macrosomia, full-term low birth weight, large for gestational age (LGA), small for gestational age (SGA), neonatal respiratory distress syndrome, and pregnancy hypertension. An infant delivered at ≥37 weeks with a birth weight of < 2500 g was considered to have a full-term low birth weight, and infants with a birth weight of ≥ 4000 g were considered to have macrosomia [27]. Neonatal respiratory distress syndrome was defined as an infant displaying an Apgar score ≤ 7 at 1 or 5 min after birth [28]. SGA refers to a standardized birth weight below the 10th percentile (P 10), and LGA refers to a standardized birth weight above P 90 [12, 19]. Premature delivery was defined as infants born before 37 weeks of gestation. Pregnant women were diagnosed with pregnancy hypertension when any abnormal blood pressure was detected (systolic blood pressure ≥140 mmHg or diastolic blood pressure ≥ 90 mmHg) from the second visit to the end of delivery [12].

### Statistical analysis

The rates of missing variables and outcomes were very low (ranging from 0.05 to 0.5%). Therefore, pregnant women for whom data for the variables/outcomes were missing were excluded from the analysis (14 of the 1820 women, 0.8%). Continuous variables (weekly GWG, maternal age, pre-pregnancy BMI and gestational weeks) are presented as the means and SD, and categorical variables (parity (1 or ≥ 2) and the age at pregnancy (≥ 35 years or < 35 years) are presented as percentages to describe the characteristics of the subjects. Because the weekly GWG is positively correlated with adverse pregnancy outcomes, such as macrosomia, LGA and pregnancy hypertension, and negatively correlated with SGA and full-term low birth weight, receiver operating characteristic (ROC) curve analyses were performed to evaluate the diagnostic value of weekly GWGs measured during the second and third trimesters in predicting the former three adverse pregnancy outcomes and preventing the latter two adverse pregnancy outcomes. A cutoff value for GWG with diagnostic significance for each adverse pregnancy outcome was selected when the integrated area under the ROC curve (AUC) was statistically significant. The lower of the cutoff values for the former three outcomes was chosen as the upper limit, while the higher cutoff value for the latter outcomes was set as the lower limit, constituting the ROC GWG targets (the ROC targets). The interquartile range (IR; range from the 25th to 75th percentiles, P25–P75) of weekly GWGs among pregnant women with GDM for whom adverse pregnancy outcomes were not recorded was calculated across the four BMI categories for Chinese women and set as the IR GWG targets (the IR targets).

Chi-squared analyses (or Fisher’s exact test if any cell was <5) were used to assess the statistical significance of difference in the incidences of adverse pregnancy outcomes across the groups of pregnant women with appropriate GWG according to the ROC, IR, and IOM targets (GWG within the targets). Considering the correlation of subjects between the groups, McNemar’s paired chi-squared tests were then performed to examine whether the incidences of adverse pregnancy outcomes in women with appropriate GWGs according to the ROC and IR targets differed from women with appropriate GWGs according to the IOM targets. Linear mixed models for repeated measures were used to account for within-subject correlations across the repeatedly measured GWGs and to study the relationship between GWG and adverse pregnancy outcomes. We then repeated linear mixed model analyses in subgroups of pregnant women who exhibited appropriate weight gain according to the ROC, IR, and IOM targets to examine whether the relationship between GWG and adverse pregnancy outcomes changed. Potential factors, such as pre-pregnancy BMI categories (underweight, normal weight, overweight, or obese), maternal age at pregnancy (≥ 35 years or < 35 years), parity (1 or ≥ 2) and gestational weeks (weeks), were included in the analytical models. Positive and negative predictive values for the cutoff value were calculated for the three targets. A Venn diagram was generated to compare the overlap of pregnant women with appropriate GWGs based on the ROC, IR, and IOM targets.

A two-tailed *P*-value < 0.05 was considered statistically significant. All data were analyzed using IBM SPSS software, version 19.0 (IBM, Armonk, NY, USA).

## Results

### Characteristics of the study population

In total, 1820 women with GDM were included in the analyses. The average age of the subjects was 29.82 (SD: 3.5) years, the average pre-pregnancy BMI was 22.14 kg/m^2^ (4.26 kg/m^2^), and the mean gestational age at GDM diagnosis and at labor were 25.9 (14.6) weeks and 39.8 (13.8) weeks, respectively. Among the subjects, 10.10% were older than 35 years, and 90.69% were primigravida. The mean weekly GWG during the second and third trimesters of pregnancy was 0.50 (0.19) kg.

### ROC and IR targets

According to the ROC curve analyses, weekly GWG in the second and third trimesters predicted pregnancy outcomes such as macrosomia, full-term low birth weight, LGA, SGA, and pregnancy hypertension (AUC ranged from 0.63 to 0.88, all *P* < 0.05) but not premature delivery or neonatal respiratory distress syndrome (data not shown). The ROC targets were 0.48–0.62 kg/week for underweight women, 0.38–0.49 kg/week for normal weight women, 0.23–0.42 kg/week for overweight women, and 0.22–0.32 kg/week for obese women (Table 1). A total of 1141 pregnant women with GDM who did not experience any adverse pregnancy outcomes were included in the analysis using the IR method. The IR targets were 0.45–0.63, 0.40–0.61, 0.29–0.53, and 0.26–0.38 kg/week for underweight, normal weight, overweight, and obese women, respectively (Table 1).

**Table 1:** Weekly GWG targets for pregnant women with GDM during the second and third trimesters according to each BMI category

Pre-pregnancy BMI (kg/m^2^)	ROC curve method^a^												IR method^b^		IOM method^c^
	*N*	Macrosomia		Full-term low birth weight		LGA		SGA		Pregnancy hypertension		Targets for GWG	*N*	Targets for GWG (P25–P75)	Targets for GWG
		AUC^c^	Cutoff value	AUC	Cutoff value	AUC	Cutoff value	AUC	Cutoff value	AUC	Cutoff value				
		(*P* value)		(*P* value)		(*P* value)		(*P* value)		(*P* value)					
<18.5	181	0.72 (0.045)	≤0.66	–	–	0.76 (0.008)	≤0.62	0.65 (0.016)	≥0.48	–	–	0.48–0.62	119	0.45–0.63	0.44-0.58
18.5–23.9	1207	0.67 (<0.001)	≤0.52	–	–	0.66 (<0.001)	≤0.62	0.63 (<0.001)	≥0.38	0.56 (0.016)	≤0.49	0.38-0.49	817	0.40–0.61	0.38–0.50
24–27.9	336	0.65 (0.003)	≤0.45	0.88 (0.003)	≥0.23	0.65 (<0.001)	≤0.42	–	–	0.58 (0.028)	≤0.44	0.23–0.42	172	0.29–0.53	0.23–0.33
≥28	86	–	–	–	–	0.64 (0.044)	≤0.32	–	–	0.69 (0.003)	≤0.40	0.22^d^–0.32	33	0.26–0.38	0.17–0.27

*GWG* gestational weight gain, *GDM* gestational diabetes mellitus, *ROC* receiver operating characteristic, *IR* interquartile range, *IOM* Institute of Medicine, *LGA* large for gestational age, *SGA* small for gestational age, *AUC* area under the curve

^a^Ten of 1820 pregnant women were excluded because of a lack of BMI data, while the remaining 1810 were included in the analysis designed to set the ROC targets setting. An ROC curve analysis was conducted to quantify the accuracy of weekly GWGs according to the pre-pregnancy BMI categories of pregnant women with GDM in predicting each adverse pregnancy outcome. The cutoff value for the weekly GWG represents the values when the AUC was statistically significant (a significant AUC means that the weekly GWG accurately predicted adverse pregnancy outcomes). Reasonable cutoff values were conservatively used as the weekly GWG targets for women in the four BMI categories. For example, in the group of women with a pre-pregnancy BMI <18.5 kg/m^2^, the cutoff values for weekly GWG for macrosomia and LGA were 0.66 and 0.62, and thus a weekly GWG of ≤ 0.66 and ≤ 0.62 helped prevent FM and LGA, respectively. Thus, 0.62 (the lower one) was selected as the upper limit. The cutoff value for weekly GWG for SGA was 0.48 and was selected as the lower limit, since greater GWG would help reduce the risk of SGA. Therefore, a weekly GWG target ranging from 0.48 to 0.62 was set as the ROC target for the group

^b^The IR method was used to calculate the weekly GWG targets (values between the 25th and 75th percentiles) among the four BMI categories for women with GDM who did not experience any adverse pregnancy outcomes (1141 pregnant women)

^c^The pre-pregnancy BMI categories for the IOM targets were <18.5 kg/m^2^, 18.5–24.99 kg/m^2^, 25–29.9 kg/m^2^, and ≥ 30 kg/m^2^ for underweight, normal weight, overweight, and obese, respectively

^d^The lower limit was calculated according to the upper limit and the range of the targets established using the IR method in the category of pre-pregnancy BMI ≥ 28 kg/m^2^ ((0.26 × 0.32)/0.38), assuming that the targets had the similar ranges

### Comparison of the incidence of adverse pregnancy outcomes

Statistically significant differences in the incidences of adverse pregnancy outcomes were not observed among the groups of pregnant women with appropriate GWGs according to the ROC, IR, and IOM targets (Table 2). However, McNemar’s chi-squared paired tests indicated a significantly lower risk of having SGA infants and pregnancy hypertension for women with appropriate GWGs according to the ROC targets than for women with appropriate GWGs based on the IOM targets (SGA, 7.5% vs. 8.6%; pregnancy hypertension, 12.6% vs. 14.1%, both *P* < 0.05). Pregnant women with GDM who had appropriate GWGs according to the IR targets had higher risks of premature delivery, pregnancy hypertension and delivering infants with macrosomia or LGA infants than the women with appropriate GWGs according to the IOM targets. However, the risk of delivering SGA infants was lower in pregnant women who had appropriate GWGs according to the IR targets than in women with appropriate GWGs based on the IOM targets (Table 2).

**Table 2:** Incidences of adverse pregnancy outcomes in women with appropriate gestational weight gain according to the ROC, IR, and IOM targets

Adverse pregnancy outcome	Pregnant women with appropriate gestational weight gain			
	ROC targets (*N*=477)	IR targets (*N*=859)	IOM targets (*N*=560)	*P* value from Chi-squared tests*
	*n* (%)	*n* (%)	*n* (%)	
Premature delivery	23 (4.8)	39 (4.5)^†^	23 (4.1)	0.85
Macrosomia	17 (3.6)	51 (5.9)^†^	22 (3.9)	0.08
Full-term low birth weight	4 (0.8)	5 (0.6)	4 (0.7)	0.86
LGA	38 (8.0)	93 (10.8)^†^	43 (7.7)	0.08
SGA	36 (7.5)^†^	69 (8.0)^†^	48 (8.6)	0.83
Neonatal respiratory distress syndrome	1 (0.2)	1 (0.1)	1 (0.2)	1.00**
Pregnancy hypertension	60 (12.6)^†^	125 (14.6)^†^	79 (14.1)	0.60

*ROC* receiver operating characteristic, *IR* interquartile range, *IOM* Institute of Medicine, *LGA* large for gestational age, *SGA* small for gestational age

*P values from chi-squared tests of differences in the incidences of adverse pregnancy outcomes among the three groups

**P values from Fisher’s exact tests

^†^*P* < 0.05 from McNemar’s chi-squared paired tests compared with the incidence of adverse pregnancy outcomes among pregnant women who exhibited appropriate weight gain according to the IOM targets

### Relationship between GWG and adverse pregnancy outcomes

After controlling for potential confounding factors, linear mixed models revealed that the GWG was significantly correlated with premature delivery (β [95% confidence interval] = –1.90 [–2.71 to –1.09]), macrosomia (2.08 [1.34~2.81]), full-term low birth weight (–2.95 [–4.61 to –1.29]), LGA (2.08 [1.51 to 2.64]), SGA (–1.66 [–2.31 to –1.00]), and pregnancy hypertension (0.75 [0.24 to 1.26]). However, a statistically significant correlation was not observed for neonatal respiratory distress syndrome (Table 3). We then repeated the analysis with the models in subgroups of pregnant women who exhibited appropriate weight gains according to the ROC, IR, and IOM targets to examine whether the three targets improved pregnancy outcomes. Statistically significant correlations between the GWG and macrosomia, full-term low birth weight, LGA, SGA, and pregnancy hypertension were not observed, while the GWG was still correlated with premature delivery (Table 3).

**Table 3:** Relationships between gestational weight gain and adverse pregnancy outcomes determined using linear mixed models^a^

Adverse pregnancy outcome	Total sample (*N*=1820)		Subgroups of pregnant women who exhibited appropriate weight gain according to the ROC, IR and IOM targets					
			ROC targets (*N*=477)		IR targets (*N*=859)		IOM targets (*N*=560)	
	Estimate (95% CI)	T (*P* value)	Estimate (95% CI)	T (*P* value)	Estimate (95% CI)	T (*P* value)	Estimate (95% CI)	T (*P* value)
Premature delivery	–1.90 (–2.71 to –1.09)	–4.60 (<0.05)	–1.70 (–2.36 to –1.04)	–5.06 (<0.05)	–1.59 (–2.24 to –0.95)	–4.82 (<0.05)	–1.24 (–1.95 to –0.53)	–3.43 (<0.05)
Macrosomia	2.08 (1.34 to 2.81)	5.56 (<0.05)	0.19 (–0.52 to 0.90)	0.52 (0.60)	0.18 (–0.37 to 0.72)	0.63 (0.53)	0.11 (–0.57 to 0.79)	0.31 (0.75)
Full-term low birth weight	–2.95 (–4.61 to –1.29)	–3.49 (<0.05)	–1.11 (–2.80 to 0.58)	–1.29 (0.20)	–1.44 (–3.20 to 0.33)	–1.6 (0.11)	–0.78 (–2.49 to 0.93)	–0.90 (0.37)
LGA	2.08 (1.51 to 2.64)	7.22 (<0.05)	0.28 (–0.20 to 0.77)	1.14 (0.25)	0.17 (–0.24 to 0.59)	0.81 (0.42)	0.22 (–0.28 to 0.72)	0.86 (0.39)
SGA	–1.66 (–2.31 to –1.00)	–4.94 (<0.05)	0.21 (–0.32 to 0.73)	0.77 (0.44)	0.29 (–0.18 to 0.76)	1.22 (0.22)	0.12 (–0.35 to 0.60)	0.51 (0.61)
Neonatal respiratory distress syndrome	–0.54 (–4.08 to 3.00)	–0.30 (0.76)	–0.29 (–3.43 to 2.85)	–0.18 (0.86)	–1.74 (–5.42 to 1.94)	–0.93 (0.35)	–2.15 (–5.39 to 1.10)	–1.30 (0.19)
Pregnancy hypertension	0.75 (0.24 to 1.26)	2.90 (<0.05)	0.24 (–0.15 to 0.63)	1.21 (0.23)	0.03 (–0.33 to 0.40)	0.17 (0.87)	–0.22 (–0.61 to 0.17)	–1.11 (0.27)

*ROC* receiver operating characteristic, *IR* interquartile range, *IOM* Institute of Medicine, *CI* confidence interval, *LGA* large for gestational age, *SGA* small for gestational age

^a^Adjusted for pre-pregnancy BMI categories (underweight, normal weight, overweight, or obese), maternal age at pregnancy (≥ 35 years or < 35 years), parity (1 or ≥ 2), and gestational weeks at labor (weeks)

### Values of the targets in predicting adverse pregnancy outcomes

The predictive values of the three targets for each adverse pregnancy outcomes were evaluated. Among the three standards, the ROC targets exhibited the best comprehensive predictive value for adverse pregnancy outcomes. The negative predictive values for macrosomia, full-term low birth weight, LGA, SGA, and pregnancy hypertension were 96.9%, 99.3%, 93.5%, 93.7%, and 87.4%, respectively. The positive predictive values were 10.1%, 3.5%, 17.3%, 17.5%, and 19.4%, respectively (Table 4).

**Table 4:** Predictive values of the ROC, IR and IOM targets for the absence of adverse pregnancy outcomes

Adverse pregnancy outcome	Target value ratio	Positive predictive value (%)			Negative predictive value (%)		
		ROC targets	IR targets	IOM targets	ROC targets	IR targets	IOM targets
Macrosomia	≥ upper: < upper	10.1	12.1	9.8	96.9	95.4	96.5
Full-term low birth weight	≤ lower: > lower	3.5	3	3.7	99.3	99.3	99.2
LGA	≥ upper: < upper	17.3	21.1	17.3	93.5	91.4	93.5
SGA	≤ lower: > lower	17.5	15.3	17.7	93.7	93.5	93.1
Pregnancy hypertension	≥ upper: < upper	19.4	21.8	19.2	87.4	85.8	87.0

*ROC* receiver operating characteristic, *IR* interquartile range, *IOM* Institute of Medicine, *LGA* large for gestational age, *SGA* small for gestational age

### Comparison of the overlap of the three targets

**Fig. 1:**
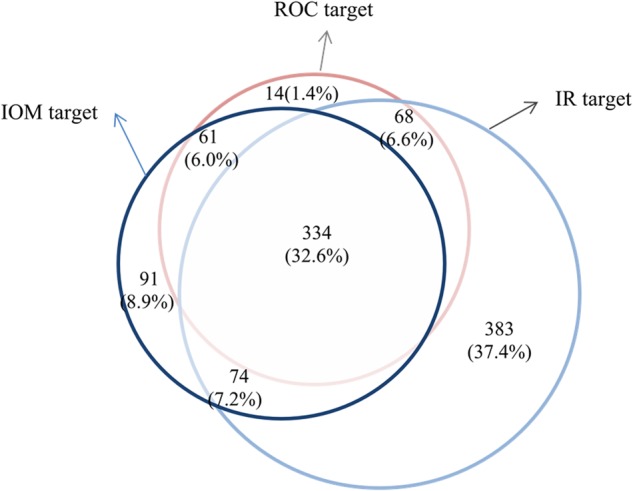
A Venn diagram showing the comparison of pregnant women with appropriate gestational weight gain based on the ROC, IR, and IOM targets

A total of 1025 pregnant women were identified as having appropriate GWGs according to at least one of the three targets, 32.6% (334) of which were shared by all three targets (Fig. 1).

## Discussion

In this retrospective cohort study of pregnant women with GDM, GWGs according to the ROC targets during the second and third trimesters improved pregnancy outcomes in women with GDM in Shanghai, China. Women with GDM with exhibiting appropriate GWGs according to the ROC targets had a lower risk of adverse pregnancy outcomes (e.g., SGA and pregnancy hypertension) than women with appropriate GWGs according to the IOM targets, and the negative predictive values of the ROC targets were superior to the IOM targets.

These findings are not consistent with the results of a previous study on Australia women, in which the authors revealed that GWGs defined according to modified stringent targets did not improve pregnancy outcomes [19]. However, the previously reported method for modifying IOM GWG targets has certain deficiencies. According to the Venn diagram, the ROC targets are more stringent than the IOM and IR targets, since the percentage of pregnant women exhibiting appropriate GWG according to the ROC targets outside the intersections of the three targets was markedly lower than the percentage defined by the IOM and IR targets (1.4%, 8.9%, and 37.4%, respectively).

Our findings are similar to the results from previous studies of pregnant Chinese women [21, 22]. The ROC targets for abnormal weight (including pre-pregnancy underweight, overweight, and obese) in women with GDM were slightly higher than the IOM targets. The differences may be attributed to the lower pre-pregnancy BMI criteria for the ROC targets than for the IOM targets and to the observation that Chinese women tend to be thinner than Western women [21, 22, 29]. Both of these aspects may increase the threshold for GWG in Chinese patients with GDM because the pre-pregnancy BMI negatively correlates with GWG [18].

GWGs for overweight and obese women with GDM defined according to the ROC targets were lower than GWGs based on the IOM targets. However, in the present study, ∼60% of the overweight and obese women with GDM experienced excessive weight gain according to the ROC targets. In addition, the increasing prevalence of overweight and obesity among women of reproductive age and a misunderstanding of GWG due to social influences and views on diet during pregnancy may result in the increasing prevalence of excessive weight gain in pregnant women who are overweight and obese [29–31]. Thus, more attention should be paid to the control of GWG in these groups of women with GDM.

GWG defined according to the IR targets was not associated with adverse pregnancy outcomes, which may be attributed to the deficiency in the method used to calculate IR targets, which was based on a relatively fixed distribution and thus lacked a rational basis for evaluating adverse pregnancy outcomes.

Our study has several limitations. First, the subjects were pregnant women who received care at the Obstetrics and Gynecology Hospital of Fudan University, which may have resulted in an inadequate representativeness of the sample population (subjects may have higher education or income levels, leading to better detection of GDM) and limited the generalizability of the results. However, given the characteristics of GDM and the large sample size (1820 cases), we consider the ROC targets as the meaningful guidelines for GWG in pregnant women with GDM. Second, weight interventions were implemented once the subjects were diagnosed with GDM but not for subjects without GDM. Thus, we were not able to compare our results to findings obtained from women without GDM. Further research is needed to understand the effects of these interventions on both GWG and adverse pregnancy outcomes. Third, the timeline of GWG assessments overlapped with the timeline of a confirmed diagnosis of pregnancy hypertension. Thus, we were not able to justify a causal relationship between these two parameters, which might have affected the conclusion regarding the association between pregnancy hypertension and GWG.

Finally, in the total sample, GWG was associated with adverse pregnancy outcomes, with the exception of neonatal respiratory distress syndrome; however, the association between GWG and pregnancy outcomes (e.g., macrosomia, full-term low birth weight, LGA, SGA, and pregnancy hypertension) was no longer statistically significant in subgroups of women with appropriate GWGs according to the ROC, IR, and IOM targets. According to the results obtained using linear mixed models, women with appropriate GWGs according to the three targets exhibited an effectively reduced risk of adverse outcomes (e.g., macrosomia, full-term low birth weight, LGA, SGA, and pregnancy hypertension), and the data did not support any of the three targets. GWG has a significant impact on adverse pregnancy outcomes, which might be enhanced in the mixed models, resulting in a decreasing effect of different targets on the outcomes. Further studies are needed to distinguish the extent of the impact of GWG itself and the GWG targets on adverse outcomes.

## Conclusions

In summary, compared with the IOM targets, ROC targets provide better GWG guidelines, which can improve pregnancy outcomes in women with GDM in Shanghai, China. Studies on the applicability of the ROC targets to pregnant women with GDM in other regions of China should be conducted to determine GDM-specific GWG targets in China.
